# A cross-sectional study of pain sensitivity, disease-activity assessment, mental health, and fibromyalgia status in rheumatoid arthritis

**DOI:** 10.1186/s13075-015-0525-5

**Published:** 2015-01-20

**Authors:** Nalinie Joharatnam, Daniel F McWilliams, Deborah Wilson, Maggie Wheeler, Ira Pande, David A Walsh

**Affiliations:** Arthritis UK Pain Centre, Division of ROD, University of Nottingham, Nottingham, UK; Department of Rheumatology, Nottingham University Hospitals NHS Trust, Nottingham, UK; Department Rheumatology, Sherwood Forest Hospitals NHS Foundation Trust, Sutton-in-Ashfield, UK; Arthritis UK Pain Centre, Academic Rheumatology, Clinical Sciences Building, City Hospital, Nottingham, UK

## Abstract

**Introduction:**

Pain remains the most important problem for people with rheumatoid arthritis (RA). Active inflammatory disease contributes to pain, but pain due to non-inflammatory mechanisms can confound the assessment of disease activity. We hypothesize that augmented pain processing, fibromyalgic features, poorer mental health, and patient-reported 28-joint disease activity score (DAS28) components are associated in RA.

**Methods:**

In total, 50 people with stable, long-standing RA recruited from a rheumatology outpatient clinic were assessed for pain-pressure thresholds (PPTs) at three separate sites (knee, tibia, and sternum), DAS28, fibromyalgia, and mental health status. Multivariable analysis was performed to assess the association between PPT and DAS28 components, DAS28-P (the proportion of DAS28 derived from the patient-reported components of visual analogue score and tender joint count), or fibromyalgia status.

**Results:**

More-sensitive PPTs at sites over or distant from joints were each associated with greater reported pain, higher patient-reported DAS28 components, and poorer mental health. A high proportion of participants (48%) satisfied classification criteria for fibromyalgia, and fibromyalgia classification or characteristics were each associated with more sensitive PPTs, higher patient-reported DAS28 components, and poorer mental health.

**Conclusions:**

Widespread sensitivity to pressure-induced pain, a high prevalence of fibromyalgic features, higher patient-reported DAS28 components, and poorer mental health are all linked in established RA. The increased sensitivity at nonjoint sites (sternum and anterior tibia), as well as over joints, indicates that central mechanisms may contribute to pain sensitivity in RA. The contribution of patient-reported components to high DAS28 should inform decisions on disease-modifying or pain-management approaches in the treatment of RA when inflammation may be well controlled.

## Introduction

Rheumatoid arthritis (RA) is a common chronic systemic inflammatory disease that causes pain and disability. Pain in active RA may reflect synovitis, but not all people with RA respond to antiinflammatory treatment, and other pain mechanisms may be important. Noninflammatory pain mechanisms in RA may include variations in central pain processing, or coincidental pathology. Pain phenotypes in patients with RA that include widespread pain, mood disturbance, and fatigue overlap with those in fibromyalgia (FM). Fibromyalgia may represent either comorbidity or a continuous phenotypic spectrum associated with variations in central pain processing [1]. Noninflammatory pain mechanisms in RA may share some common characteristics with those found in other arthropathies, such as osteoarthritis (OA), in which greater sensitivity to pain at nonjoint sites, as well as at sites affected by arthritis, may indicate augmented central pain processing [2]. Previous studies have identified and partially characterized people with RA who fulfill classification criteria for FM [3]. In general, people with RA and FM show higher pain, higher measures of disease activity, and poorer mental health, but also less structural damage [4,5]. Psychological distress may be associated with greater pain sensitivity [6].

The 28-joint disease activity score (DAS28) is commonly used in RA as a clinical measure of inflammatory disease activity and to guide treatment decisions [7,8]. It appears that higher pain levels contribute to higher DAS28 scores [9,10]. DAS28 combines four discrete components into an overall measure of RA inflammatory disease activity; a 28-swollen joint count (SJC), 28 tender-joint count (TJC), acute-phase response (commonly the erythrocyte sedimentation rate (ESR)), and a general health assessment using a visual analogue score (VAS-GH). Of these components, SJC and acute-phase response are observed by the assessor, and VAS and TJC are reported by the patient. DAS28-P is a derived index for the contribution of patient-reported components to the DAS28, which, we have proposed, may provide a quantitative assessment of noninflammatory contributions to pain in early RA. A high DAS28-P was associated with worse pain and disability in early RA [11], and DAS28-P may be high in people with FM [12]. Tenderness may be associated with augmented pain processing and other features of FM in RA, and DAS28 scores may overestimate inflammatory disease activity in people with a high TJC [12,13]. The DAS28-P index is currently a research tool and has not been validated previously as a measure of augmented pain in people with long-standing RA.

Quantitative sensory testing can be used to assess pain mechanisms [14]. Pressure pain-detection thresholds (PPTs) measure the intensity required for blunt pressure stimuli to produce pain. Differences in PPTs between individuals at sites distant to tissue injury may indicate augmented central pain processing [2]. In this study, we hypothesized that, in people with RA on stable disease-modifying treatment, pain sensitivity, measured by lower PPTs, is associated with a high contribution of patient-reported factors to DAS28 and with features of FM. Associations between higher DAS28-P indices and pain sensitivity or FM would support interpretation of DAS28-P as a measure of augmented pain processing. We also aimed to explore, within a single patient group, associations between pain sensitivity, FM classification, mental health, and their effects on disease-activity assessment.

## Methods

A consecutive sample of 50 participants with RA of >2-year duration, who reported current pain and displayed DAS28 > 3.1 [11], were recruited to participate in a study of pain in RA from a rheumatology outpatients clinic at Sherwood Forest Hospitals NHS Foundation Trust during a 3-week period in 2013. Written, informed consent was obtained as per the Declaration of Helsinki. Ethical approval for this project was obtained from the East Midlands NREC–Nottingham 2 (13/EM/0047). The American College of Rheumatology/European League Against Rheumatism criteria 2010 [15] were used to determine RA classification.

### Clinical assessment

Demographic data, age, gender, ethnicity, smoking status, current drug regimen, and recent analgesia were collected at examination. The DAS28-ESR was derived from clinical assessment by a single observer (NJ) who was blinded to questionnaire responses.

Participants completed a questionnaire set for pain status and other patient-reported characteristics. These questionnaires were chosen because they have been used in previous research to measure pain experience, beliefs, and emotional status. The set consisted of Short Form McGill Pain Questionnaire [16]; Intermittent and Constant Osteoarthritis Pain (ICOAP) [17]; Beck Depression Inventory (BDI) [18]; State-Trait Anxiety Index short form (STAI-SF) [19]; Fatigue Severity Scale (FSS) [20]; Widespread Pain Index (WPI) [21]; Symptom Severity Scale (SSS) [22]; and a 0 to 10 numerical rating scale (NRS) for current knee pain. WPI and SSS questionnaires were included to permit FM classification according to the American College of Rheumatology criteria 2010 [22]. A summation of the WPI and SSS questionnaire scores was used as a measure of fibromyalgia [23].

### Pressure-pain detection thresholds

Participants undertook quantitative sensory testing for pressure-pain detection thresholds (PPT), by using an electronic pressure algometer with a laptop recording/display device and a patient switch (Somedic Sensebox). It was carried out by a single, trained observer (NJ) at the medial tibiofemoral joint line of the most painful knee, or the right knee if no pain existed in either, and also at sites distal to (anterior tibia) and distant from (sternum) the index knee. Knee pain is a common source of disability in people with RA, and PPT assessment has been standardized by us and others at these sites in people with OA [2,24], permitting comparison between conditions.

Participants were first familiarized with the procedure by applying the stimulus to a learning site on one hand. PPTs were then measured sequentially at sternum, medial knee, and anterior tibia. For each test, the pressure (kPa) was recorded at which pain was first experienced during application of progressively increasing pressure by using a probe with a 1 cm^2^ blunt end and a constant ramp of 50 kPa per second [25]. The test stimulus was applied to each site/3 times with 1-minute intervals between repeats and between sites.

### Statistics

The mean of the three PPT values for each site was log transformed to ensure normality before analysis. For the anterior tibia, the mean of the second and third PPT values was used, because pilot analyses indicated that measurement 1 was significantly lower than measurements 2 and 3. Normal distributions after transformation were confirmed by Kolmogorov-Smirnov tests. DAS28-P was derived from the components of the DAS28 scores, as previously reported [11], by using a rearrangement of the DAS28-ESR formula; DAS28-P = (0.56 × √TJC + 0.014 × VAS-GH)/DAS28. Pearson (parametric) or Spearman (nonparametric) correlations were used to assess the association between continuous variables, and Student *t* tests (parametric) or Mann–Whitney *U* tests (nonparametric) were used to determine differences between groups. Univariate test results are presented without adjustments for multiple comparisons. Multivariable regression models were used to adjust for predefined confounding variables during analyses. Multiple linear regression was used with continuous outcome variables (for example, log-transformed PPT) and binary logistic regression, with all co-variables entered simultaneously, calculated adjusted odds ratios (aOR).

For logistic regression, the continuous independent variables were divided into tertiles. Each regression model included age and gender to adjust for common confounders. The statistical software used was the Statistical Package for Social Scientists (SPSS) v. 21, and statistical significance was taken as *P* < 0.05.

## Results

Participant demographics, clinical measures, PPT, and drug regimens are shown in Table 1. The majority of participants were using analgesics, and all were using at least one traditional or biologic DMARD and/or glucocorticoid. No participant had used tricyclic antidepressants, gabapentin, or pregabalin within 24 hours before assessment. Current knee pain (NRS > 0) was reported by 40 (80%) participants. All participants had DAS28 scores >3.1, consistent with active RA [7], and eight participants (16%) displayed high disease activity (DAS28 > 5.1). Data from PPT, DAS28-P, DAS28, and all questionnaires (except for knee pain) did not significantly diverge from normal distributions. DAS28 scores were higher in those currently using biologics (biologics mean (SD) = 5.5 (1.0) compared with nonbiologics (mean (SD) = 4.6 (0.9); *P* < 0.05), and in those using steroids (steroids mean (SD) = 5.5 (0.9) compared with no steroid mean (SD) = 4.5 (0.8), *P* < 0.05).

**Table 1:** **Participant characteristics**

		**Whole study group**	**Fibromyalgia classification**	
		**Median (IQR) or %**	**Yes**	**No**
**N**		50	24	26
**Female**		76%	75%	77%
**Age**		60 (54–69)	58 (54–66)	63 (54–70)
**Current smoker**		21%	26%	17%
**Analgesia**	Any	69%	71%	65%
	Paracetamol	63%	71%	54%
	NSAID	16%	17%	15%
	Opiate	37%	38%	35%
**DMARD**	Methotrexate	59%	50%	65%
	Sulfasalazine	22%	15%	25%
	Other nonbiologic DMARD	36%	40%	30%
	Combination nonbiologic DMARD	24%	29%	15%
**Biologic therapy**		12%	8%	16%
**Combination therapy**		31%	33%	27%
**Glucocorticosteroid**		20%	15%	21%
**Pressure pain threshold**				
	Knee	173 (119–287)	133 (58–181)**	239 (169–470)
	Tibia	140 (98–246)	108 (61–167)**	187 (122–337)
	Sternum	155 (85–211)	111 (70–175)**	179 (149–256)

Demographics of the entire study group and a comparison between participants that did or did not satisfy fibromyalgia-classification criteria. Other nonbiologic DMARDs were azathioprine (*n* = 2), leflunomide (*n* = 4), gold (*n* = 3), hydroxychloroquine (*n* = 6), and cyclosporin (*n* = 3). Biologic therapies were antitumor-necrosis factor agents (n=4), rituximab (n=1), or abatacept (n=1). Combination DMARDs; two or more biologic or nonbiologic DMARDs. **P < 0.01 (compared with participants who did not satisfy criteria for fibromyalgia).

### Pain-pressure thresholds

PPT values are shown in Table 1. PPTs were lower (more sensitive) at the anterior tibia and sternum than at the medial tibiofemoral joint line (*P* = 0.007 and *P* = 0.005, respectively), but PPT at each site was significantly correlated with PPT at each other site (r > 0.64, *P* < 0.001 for all associations). Low PPT at each site was associated with higher reported pain, irrespective of the site or aspect of pain addressed by the questionnaire (Table 2).

**Table 2:** **Associations of clinical characteristics with pressure pain-detection thresholds**

			**Medial knee**	**Tibia**	**Sternum**
**DAS28**			−0.62**	−0.62**	−0.60**
		TJC	−0.53**	−0.50**	−0.48**
		VAS-GH	−0.52**	−0.46**	−0.52**
		SJC	−0.04	0.03	−0.04
		ESR	−0.12	−0.21	−0.14
**DAS28-P**			−0.46**	−0.41**	−0.41**
**Pain**	McGill Pain Questionnaire	Continuous	−0.43**	−0.60**	−0.47**
		Intermittent	−0.41**	−0.41**	−0.40**
		Neuropathic	−0.45**	−0.49**	−0.38**
		Affective	−0.30*	−0.43**	−0.33*
		**Total score**	−0.47**	−0.57**	−0.46**
	ICOAP	Constant	−0.34**	−0.39**	−0.35*
		Intermittent	−0.35*	−0.56**	−0.41**
	Knee pain		−0.53**	−0.49**	−0.46**
	Widespread Pain Index		−0.36*	−0.40**	−0.33*
**Fatigue**	Fatigue Severity Scale		−0.27	−0.33*	−0.27
**Mood**	Beck Depression Index II		−0.40**	−0.35*	−0.36*
	State-Trait Anxiety Index		−0.24	−0.16	−0.12
**Somatic symptoms scale**			−0.28*	−0.30*	−0.27*
**Fibromyalgia**			−0.44**	−0.41**	−0.39**

Negative values indicate that higher levels of trait are associated with lower PPTs (more sensitive). Unadjusted correlation coefficients for log-transformed PPT are shown. Fibromyalgia is calculated from Widespread Pain Index + Somatic Symptoms Scale. **P < 0.01, *P < 0.05 for significance of association.

Lower PPT (more sensitive) at each site was also associated with higher DAS28 and DAS28-P (Table 2). DAS28-P and DAS28 were positively correlated (r = 0.73; *P* < 0.001) (Table 1), which had the potential to confound analyses strongly. Each component of the DAS28 score was therefore examined for association with PPTs. Associations between PPT and DAS28 were attributable to significant associations with the patient-reported components of DAS28 (VAS-GH and tender-joint count), whereas significant associations were not detected with ESR or swollen-joint counts, both in univariate (Table 2) and in multivariable analyses that included all four of theDAS28 components (Table 3). Lower PPTs were also associated with low mood or somatic symptoms, but were not significantly associated with reported anxiety (Table 2), gender or age, nor with analgesic (including opioid) or combination DMARD use (data not shown).

**Table 3:** **Multivariable associations of pressure-pain detection thresholds (PPTs) with DAS28 components**

**DAS28 component**		**Medial knee**		**Tibia**		**Sternum**	
		**β**	***P***	**β**	***P***	**β**	***P***
**Patient-reported**	TJC	−0.37	0.030	−0.42	0.017	−0.29	0.092
	VAS-GH	−0.33	0.047	−0.22	0.195	−0.38	0.029
**Observed**	SJC	−0.08	0.539	−0.03	0.842	−0.05	0.719
	ESR	−0.07	0.614	−0.22	0.134	−0.10	0.950

Standardized β values and *P* values (not corrected for multiple comparisons) are shown for three regression models comparing the influence of DAS28 components on PPT. Multiple linear regression models with log-transformed PPT at each testing site as dependent variable, and TJC, VAS-GH, SJC, ESR, age, and gender as predictor variables.

### Fibromyalgia classification and fibromyalgia

Twenty-four (48%) participants satisfied FM classification criteria based on responses to WPI and SSS questionnaires. Participants displayed similar demographic characteristics and drug use whether or not they satisfied FM classification criteria (Table 1). Participants who satisfied FM classification criteria displayed lower PPT at each site (Table 1). Associations between PPT and FM classification were confirmed in logistic regression analyses (aOR (95% CI) for tertiles of either the anterior tibia PPT, 0.4 (0.2 to 0.9), *P* = 0.035; the medial knee joint line 0.2 (0.1 to 0.5), *P* = 0.001; or the sternum 0.3 (0.1 to 0.7), *P* = 0.007).

Participants who satisfied FM classification criteria also displayed higher DAS28 or DAS28-P (Table 4). Associations were attributable to significant univariate associations with the patient-reported components of DAS28 (VAS-GH and tender-joint count) but not ESR or swollen-joint counts (Table 4). Logistic regression using all four DAS28 components confirmed that VAS-GH was significantly associated with FM classification (aOR (95% CI) 3.7 (1.4 to 9.5), *P* = 0.008), whereas TJC, SJC, and ESR were not (aOR (95% CI) 1.5 (0.6 to 4.1), *P* = 0.403, 1.1 (0.5 to 2.6), *P* = 0.789 and 0.8 (0.4 – 1.9), *P* = 0.673, respectively).

**Table 4:** **Associations of clinical characteristics with fibromyalgia**

			**Fibromyalgia classification**		**Fibromyalgia**	
			**Yes**	**No**	**r**	***P***
**DAS28**			4.8 (4.4 – 5.3)**	4.4 (3.8 – 4.9)	0.46	0.001
		TJC	11 (7 – 18)**	6 (4 – 9)	0.35	0.012
		VAS-GH	70 (55 – 78)**	42 (24 – 55)	0.60	<0.001
		SJC	1 (0 – 2)	1 (1 – 2)	−0.03	0.816
		ESR	19 (8 – 29)	17 (12 – 26)	0.01	0.963
**DAS28-P**			0.58 (0.52 – 0.64)**	0.50 (0.45 – 0.57)	0.43	0.002
**Pain**	McGill Pain Questionnaire	Continuous	5.3 (3.2 – 6.7)**	2.3 (1.8 – 3.8)	0.49	<0.001
		Intermittent	4.0 (3.0 – 5.5)**	1.7 (0.7 – 2.8)	0.61	<0.001
		Neuropathic	3.2 (2.3 – 5.7)**	1.5 (0.8 – 3.8)	0.54	<0.001
		Affective	4.0 (2.5 – 6.5)**	1.8 (0.3 – 4.0)	0.53	<0.001
		**Total score**	3.8 (3.2 – 5.8)**	2.2 (1.1 – 3.1)	0.65	<0.001
	ICOAP	Constant	42 (8 – 65)**	8 (0 – 35)	0.45	0.001
		Intermittent	42 (29 – 58)**	8 (0 – 32)	0.57	<0.001
	Knee pain		7 (5 – 8)**	3 (0 – 6)	0.50	<0.001
	Widespread Pain Index		10 (7 – 13)**	4 (2 – 4)	0.86	<0.001
**Fatigue**	Fatigue Severity Scale		52 (38 – 61)*	39 (25 – 57)	0.46	0.001
**Mood**	Beck Depression Index II		16 (10 – 30)*	13 (8 – 18)	0.43	0.003
	State-Trait Anxiety Index		0 (^−^6 – 3)**	^–^6 (^–^9 – ^–^2)	0.39	0.006
**Somatic symptoms scale**			9 (7–10)**	4 (3 – 7)	0.83	<0.001

Differences between DAS28, DAS28-P, individual score components, and mental health questionnaire scores for participants who did or did not satisfy fibromyalgia criteria, plus correlations of these variables with fibromyalgia, calculated from the summated Widespread Pain Index and Somatic Symptoms Scale scores. *P < 0.05, **P < 0.01 for differences between groups, or associations.

**Figure 1:**
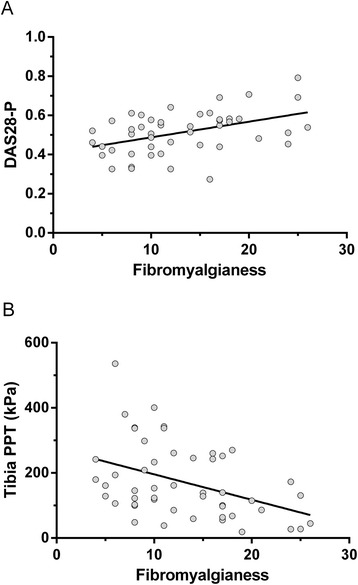
**Scatter plots of (A) DAS28-P indices and (B) anterior tibial PPT measurements versus fibromyalianess score (WPI + SSS).** Best-fitted linear regression lines for each plot are shown. Other PPT measurements and the patient-reported components of DAS28 all showed similar plots.

The summated (WPI + SSS) measure of fibromyalgia had a mean (SD) of 13 (6), a median (IQR) 12 (8 to 17), and a range from 4 to 26. DAS28 and DAS28-P were each positively correlated with WPI + SSS (Figure 1A; Table 4). Associations with fibromyalgia were attributable to significant associations with questionnaire scores in each of WPI (DAS28, r = 0.43, *P* = 0.002, and DAS28-P r = 0.43, *P* = 0.003) and SSS (DAS28 r = 0.34, *P* = 0.019, and DAS28-P r = 0.32, *P* = 0.028), and due to significant associations of the patient-reported components of DAS28 (VAS-GH and tender-joint count), but not ESR or swollen-joint counts (Table 4). For the WPI, correlation coefficients (*P* values) for TJC, VAS-GH, SJC, and ESR were 0.33 (*P* = 0.02); 0.55 (*P* < 0.001); 0.01 (*P* = 0.94) and 0.00 (*P* = 0.99), respectively. For SSS, correlation coefficients (*P* values) for TJC, VAS-GH, SJC, and ESR were 0.24 (*P* = 0.09); 0.47 (*P* = 0.001); −0.04 (*P* = 0.81), and 0.09 (*P* = 0.56), respectively. Higher fibromyalgia score was associated with lower PPTs (Table 2, Figure 1B), attributable to significant associations with the WPI (r values < −0.35 to −0.42, each *P* < 0.012) and SSS (r values < −0.24 to −0.32, each *P* < 0.089; statistically significant at two of three test sites) questionnaire scores. Participants who fulfilled FM criteria or had higher fibromyalgias also scored higher for pain, anxiety, and fatigue, and reported lower mood (Table 4).

Multiple regression analyses confirmed that DAS28-P was associated with FM classification (aOR (95% CI) 2.8 (1.2 to 6.4), P = 0.014) and with the summated (WPI + SSS) measure of fibromyalgia (standardized β = 0.50, *P* = 0.001), after adjustment for age and gender in both models.

## Discussion

We provide evidence that augmented pain processing is associated with pain and distress in people with established RA, and confounds assessment of inflammatory disease activity when using DAS28. We also show that DAS28-P has potential to be used as a marker for augmented pain processing in people with established RA during stable treatment with disease-modifying agents.

Low PPTs (more sensitive) were associated with greater reported pain in patients with established RA. Low PPTs have been associated with higher reported pain in other chronically painful musculoskeletal conditions, including osteoarthritis [14] and FM [26]. Low PPTs are also associated with neuropathic pain characteristics, as determined by questionnaires [27,28], and we confirmed this association in people with RA by using the neuropathic scale of the McGill Pain Questionnaire. However, similar associations were found with other pain characteristics, suggesting that low PPTs may be a feature of chronic musculoskeletal pain in general, rather than indicating specifically neuropathology.

We found that PPTs over joints were correlated with PPTs at sites distant to joints, and associations between PPTs and reported pain, mental health, and fatigue were equally strong at both articular and nonarticular sites. These findings suggest that PPTs reflect general patient-level characteristics, rather than only peripheral sensitization in inflamed joints. Similarly, widespread reductions in PPTs in people with OA [14] or FM [26] have been taken to indicate augmented central pain processing, and sensitivity to pressure has been associated with increased activity in several brain regions during functional MRI studies [29,30]. PPTs are lower in people with RA than in controls [31] and generalized hyperalgesia and allodynia occur in RA [32]. These and our data indicate that low PPTs may provide evidence of augmented central pain processing in people with RA.

We found that a high proportion (48%) of patients attending secondary care follow-up with established RA satisfied FM-classification criteria. Others have also found a higher prevalence of FM in people with RA than would be expected in the normal population [33]. In one large U.S. longitudinal cohort study, approximately 10% of participants with RA satisfied FM criteria at one time point, and close to 20% satisfied FM criteria at some point during follow-up [3]. Our participants were selected on the basis of DAS28 > 3.1 and current pain, despite receiving stable treatment with traditional or biologic DMARDs, and were largely attending secondary care appointments for DMARD monitoring or annual review. People with worse pain may be more likely to volunteer for studies of pain. The prevalence of FM in our study may well reflect its specific recruitment context. Overlap between features of FM (fatigue and somatic symptoms) and RA may lead to overclassification of FM in people with RA, although these overlapping features may also reflect common underlying mechanisms, or a subgroup of people with established RA that is prone to augmented pain.

FM is itself a condition with augmented pain processing, identified by quantitative sensory testing and functional MRI [34]. Consistent with this, we found that participants with RA who satisfied FM criteria also exhibited lower PPTs than did those who did not. Associations were found between low PPTs and worse scores on questionnaires addressing a range of FM characteristics, including widespread pain, somatic symptoms, fatigue, and low mood. Each of these questionnaires produced data that did not significantly diverge from normal distributions, and the WPI and SSS, plus their summated total, were each associated with PPTs and other fibromyalgic features. These data are consistent with the proposal that fibromyalgia represents a continuous trait rather than a dichotomous comorbidity in people with RA, and that low PPT is a component of fibromyalgia in RA.

One sixth of our participants displayed DAS28 > 5.1, suggesting high disease activity, and the remainder satisfied DAS28 criteria for active RA. High DAS28 scores in this patient group were largely determined by high VAS-GH and TJC, and high TJCs can lead to overestimation of inflammatory disease activity in RA [10]. Lower PPTs were associated with high VAS-GH and TJC, but not with ESR or SJC, indicating that augmented pain processing may be associated with inflated DAS28 in people with well-controlled inflammatory disease. This confounding of DAS28 scores was closely associated with other features of FM.

We have previously shown that DAS28-P, the proportion of DAS28 attributable to the patient-reported components VAS-GH and TJC, is associated with current pain, and predicts poor pain prognosis in people with early RA [11]. The current study further indicates associations between DAS28-P and pain in RA of more than 2 years’ duration, and supports our hypothesis that DAS28-P may reflect augmented central pain processing in people with RA.

Participants in this study were attending for annual review or DMARD monitoring, and the low observed prevalence of swollen joints and elevated ESR was consistent with well-controlled inflammation. Where swollen joint counts and ESR contribute little to DAS28, variations in tender-joint counts and VAS-GH may have similar effects on both DAS28 and DAS28-P. In early RA, where inflammation was often incompletely controlled, we demonstrated only weak associations between DAS28-P and DAS28 [11], whereas in the current study, DAS28-P was strongly associated with DAS28 (r = 0.73). Analysis of DAS28 components confirmed that associations of DAS28 with low PPTs and fibromyalgia were attributable to the patient-reported DAS28 components. Our data indicate that, for people with RA in whom inflammatory disease is well controlled, DAS28 itself may predominantly reflect augmented pain processing, and should be used with great caution as a measure of inflammatory disease activity.

Joint inflammation may itself drive augmented central pain processing [35], and RA therefore may contribute to the development of FM [3]. However, associations of FM with poorer mental health and with fatigue, in both RA and non-RA populations, indicate that factors in addition to synovitis may be associated with the development of augmented pain processing and fibromyalgia in RA. Our data indicate that changes in pain processing may persist after synovitis has settled, but further research is required to determine possible contributions of central processing to pain in people with active synovitis. The lack of significant associations between SJC or ESR and PPT suggests that ongoing inflammation was not the predominant drive for pain sensitivity in these patients, but interventional studies would be required to determine whether more intensive suppression of inflammation in early arthritis could reduce the contribution of augmented processing to pain even after inflammation is controlled.

Our research has several limitations, being a cross-sectional study of an RA population on stable treatment within a secondary-care context. Fibromyalgia can affect many aspects of life quality on which data were not collected in the current study, including sleep disturbance and loss of concentration/memory. Additional factors not anticipated in our study may have confounded some of our findings.

The use of 28 swollen-joint counts and ESR to measure inflammation in RA also has limitations. ESR might be within the normal range in people with active RA [36], and DAS28 does not address feet, which might be a site of synovitis. Future studies could use ultrasound or magnetic resonance imaging to determine whether subclinical inflammation might contribute to pain or reduced pain-detection thresholds in people with RA, and whether more-intensive immunosuppression might further improve pain outcomes. Participants were using a range of analgesic medications, which may have affected pain reporting and PPTs, although *post hoc* analyses did not reveal any association between analgesic use and PPTs in our study group. Our findings may be limited to the specific PPT protocol used, including sequence of test sites, and further research would be required to determine whether conclusions can be generalized to other quantitative sensory testing modalities, such as thermal pain thresholds.

It is unclear from our cross-sectional study whether augmented pain processing exacerbates pain in RA, whether chronic pain, as experienced in RA, leads to changes in pain processing, or whether confounding factors contribute to the observed associations. It is likely that altered pain processing may both contribute to and be a consequence of joint pathology. Associations of pain and PPT with FM measurement, both in the current and in previous [10,13] studies, additionally support the concept that comorbidity may contribute to both pain severity and reduced pain thresholds in RA. Further prospective research is merited to explore whether and which PPT methodologies could be used to identify a subgroup of people with RA who may benefit from treatments that target pain processing. Aspects of procedure, equipment, and cut-off PPT values may each affect the validity of PPT in defining patient subgroups.

Pain remains a major problem for people with RA, and we have shown that pain remains problematic despite inflammatory disease control with traditional and biologic DMARDs [11]. Our study confirms interrelations that have been inferred from previous studies between augmented pain, mental health, fibromyalgic features, and measures of RA disease activity [4,5,13,37]. Augmented pain processing may be associated with poor pain prognosis in people treated with traditional antiinflammatory strategies, especially in people with high DAS28-P [11]. Further escalation of traditional or biologic DMARD is unlikely to control the pain and distress associated with augmented central pain processing, but may result in important adverse events. Other pharmacologic, psychological, and physiotherapeutic approaches may be helpful for people with augmented pain processing associated with other forms of chronic musculoskeletal pain, such as FM or low back pain [38-40], and should continue to be investigated in people with RA [41].

There is potential for DAS28-P, individual DAS28 components, or PPTs to offer stratification tools for selecting patients for specific treatments, but their potential should first be examined in larger, generalizable populations and validated in prospective studies. At present, clinicians should consider both inflammatory disease activity and augmented central pain processing as potential mediators of pain, and plan treatment based on holistic assessment in partnership with patients with RA.

## Conclusions

- People with established RA commonly satisfy fibromyalgia classification criteria, and these people report poorer mental health and display increased pain sensitivity to pressure at nonarticular sites.
- Fibromyalgic symptoms, sensitivity to pain, and poorer mental health are all associated with increased DAS28 scores
- High disease-activity scores in some people with RA may reflect augmented pain processing rather than active inflammatory disease.
- Pain in RA should be managed alongside inflammatory disease activity, as noninflammatory pain mechanisms are prevalent, and suppression of inflammation may not eliminate pain.

## Abbreviations

BDI: Beck Depression Inventory
DAS28: 28-joint Disease Activity Score
DMARD: Disease-modifying Anti-rheumatic Drug ESR, erythrocyte sedimentation rate
FM: fibromyalgia
FSS: Fatigue Severity Scale
ICOAP: intermittent and constant osteoarthritis pain
MRI: magnetic resonance imaging
NRS: 0 to 10 numeric rating scale
PPT: pain pressure threshold
QST: Quantitative Sensory Test
RA: Rheumatoid arthritis
SJC: 28-swollen joint count
SSS: Symptom Severity Scale
STAI-SF: State-Trait Anxiety Index. short form
TJC: 28-tender joint count
VAS-GH: Visual Analogue Score for general health
WPI: Widespread Pain Index
